# “Best Practice” Skills Lab Training vs. a “see one, do one” Approach in Undergraduate Medical Education: An RCT on Students’ Long-Term Ability to Perform Procedural Clinical Skills

**DOI:** 10.1371/journal.pone.0076354

**Published:** 2013-09-25

**Authors:** Anne Herrmann-Werner, Christoph Nikendei, Katharina Keifenheim, Hans Martin Bosse, Frederike Lund, Robert Wagner, Nora Celebi, Stephan Zipfel, Peter Weyrich

**Affiliations:** 1 Department of Psychosomatic Medicine and Psychotherapy, University Hospital of Tübingen, Tübingen, Germany; 2 Department of General Internal Medicine and Psychosomatics, University Hospital of Heidelberg, Heidelberg, Germany; 3 Department of General Paediatrics, Neonatology and Child Cardiology, University Hospital of Düsseldorf, Düsseldorf, Germany; 4 Department of Anaesthesiology, University Hospital Heidelberg, Heidelberg, Germany; 5 Department of Diabetes, Endocrinology, Angiology, Nephrology and Clinical Chemistry, University Hospital of Tübingen, Tübingen, Germany; University of Minho, Portugal

## Abstract

**Background:**

Benefits of skills lab training are widely accepted, but there is sparse research on its long-term effectiveness. We therefore conducted a prospective, randomised controlled-trial to investigate whether in a simulated setting students trained according to a "best practice" model (BPSL) perform two skills of different complexity (nasogastral tube insertion, NGT; intravenous cannulation, IVC) better than students trained with a traditional "see one, do one" teaching approach (TRAD), at follow-up of 3 or 6 months.

**Methodology and Principal Findings:**

94 first-year medical students were randomly assigned to one of four groups: BPSL training or TRAD teaching with follow-up at 3 (3M) or 6 (6M) months. BPSL included structured feedback, practice on manikins, and Peyton’s "Four-Step-Approach", while TRAD was only based on the "see one - do one" principle. At follow-up, manikins were used to assess students’ performance by two independent blinded video-assessors using binary checklists and a single-item global assessment scale. BPSL students scored significantly higher immediately after training (NGT: BPSL3M 94.8%±0.2 and BPSL6M 95.4%±0.3 percentage of maximal score ± SEM; TRAD3M 86.1%±0.5 and TRAD6M 84.7%±0.4. IVC: BPSL3M 86.4%±0.5 and BPSL6M 88.0%±0.5; TRAD3M 73.2%±0.7 and TRAD6M 72.5%±0.7) and lost significantly less of their performance ability at each follow-up (NGT: BPSL3M 86.3%±0.3 and TRAD3M 70.3%±0.6; BPSL6M 89.0%±0.3 and TRAD6M 65.4%±0.6; IVC: BPSL3M 79.5%±0.5 and TRAD3M 56.5%±0.5; BPSL6M 73.2%±0.4 and TRAD6M 51.5%±0.8). In addition, BPSL students were more often rated clinically competent at all assessment times. The superiority at assessment after training was higher for the more complex skill (IVC), whereas NGT with its lower complexity profited more with regard to long-term retention.

**Conclusions:**

This study shows that within a simulated setting BPSL is significantly more effective than TRAD for skills of different complexity assessed immediately after training and at follow-up. The advantages of BPSL training are seen especially in long-term retention.

## Introduction

The skills lab is an established part of a medical faculties’ training programme. It offers a protected, “mistake forgiving” training environment [1] that allows students to practice procedures on manikins, with standardized patients or with each other prior to performing procedural skills on real patients [2-4]. Skills lab trainings have shown to improve procedural skills in novices as well as experts [5-8]. This applies to complex surgical skills [8] as well as basic clinical skills performed by medical students [9]. Furthermore there seems to be evidence, that simulation-based medical education (SBME) positively influences the outcome in the clinical setting [10,11].

In a systematic review, Issenberg and colleagues describe aspects that influence the effectiveness of SBME [5]. The key factor seen is educational feedback, providing a chance for reflection on procedural performance. Other elements including “deliberate practice”, “integration into curriculum” and “validity of simulators” also contribute to the significant success of SBME. However, not much is known about the long-term retention of procedural skills acquired during SBME, although practical proficiencies are known to abate over time, if they are not repeatedly practised [12].

In general, theoretical knowledge seems to be retained better than practical skills, and the performance of simpler tasks seems to be lost more slowly than complex ones [13,14]. The majority of studies looking at the long-term retention of procedural skills focus on techniques in basic and advanced cardiac life support training. In this setting, a significant decline in performance could be shown to start as early as a couple of weeks after initial training or could begin up to a year later. The most significant decline occurred between 6 and 12 months of time [15-18]. The effectiveness and retention of other skills taught in the SBME setting have been studied less, and much heterogeneity is seen with regards to performed skills, study subjects and teaching methods, rendering data interpretation difficult. Examples include competencies in laparoscopic surgery or colonoscopy by surgical residents after 3 months [13,19], a significant decay in temporary haemodialysis catheter insertion skills by nephrology fellows after 6 months [20] and a satisfactory retention of a rare but crucial procedural skill like coniotomy performed by trained anaesthetist up to one year [21]. This heterogeneity in findings makes it hard if not impossible to draw any conclusions for skills lab training in medical undergraduates. In summary, our current understanding of factors contributing to long-term retention of SBME trained skills is still limited owing to general data shortage, flaws in study design (heterogeneity in training methods, number of redundant practice, etc.) and heterogeneity in tested skills with regards to their complexity.

Within a SBME setting, different teaching components comprise the “best practice” skills lab training. Amongst it are feedback and repetitive practice as key factors of effective SBME [5], and instruction methods like Peyton’s “Four-Step-Approach” which seems to provide a reliable and yet quite popular teaching method [22]. In this respect, it was implemented as standard instruction for resuscitation courses of the European Resuscitation Council [23]. There is, however, conflicting evidence whether skills lab teaching following a “best practice” approach (BPSL) leads to a better performance than other established teaching methods, for example a more traditional teacher-centred “see one, do one” approach (TRAD), which is a main component of clinical bedside teaching [24]. In this form of teaching, students learn by merely watching an experienced doctor explaining and demonstrating the skill [25]. The expert acts as a role model and the first independent performance of procedural clinical skills is already with a real patient. Two recent studies could show that skills lab training following a “best practice” model with structured individual feedback, practice on manikins and Peyton’s “Four-Step-Approach” is superior to traditional bedside teaching immediately after teaching [9]. However, these findings are solely based on performance assessments immediately after the respective teaching, and research comparing long-term effects is still lacking. Knowledge about long-term retention is crucial though, as medical students often experience a time lapse between their skills lab training and actual performance on patients. This happens even more so, since the importance of early clinical teaching in the pre-clinical phase has been stressed more intensively throughout many curricula worldwide [26,27]. In light of limited resources and an already high study load there is only limited capacity for repetitive classes. Hence, there is a clear need for established methods for effective SBME providing a maximum of retention.

To our knowledge, so far there has been no randomized and prospective study investigating the effect of two different teaching approaches for undergraduate medical students for skills of different complexity with regards to long-term outcomes. We therefore investigated the effects of two different teaching methods within a simulated setting on the long-term performance of undergraduate medical students: a “best practice” example of skills lab training (BPSL) incorporating structured individual feedback, practice on manikins and Peyton’s “Four-Step-Approach” vs. a traditional “see one, do one” approach (TRAD) similar to bedside teaching. As task complexity is an important variable with regards to skills retention over time [14,19], we have chosen two skills with different complexity level for investigation, namely nasogastric tube insertion, NGT, as a simpler and i.v. cannulation, IVC, as a more complex procedure. As the time lapse between training and assessment is another important variable for retention, we assessed the students’ performance at 3 and 6 months respectively, resulting in a study design comprising four independent arms. Our data support a “best practice” form of SBME (BPSL) to be more effective than formerly used “see one, do one” approaches (TRAD), especially for the long-term retention of trained clinical skills with higher manual complexity.

## Methods

### Study design

We conducted a randomised controlled trial to investigate the long-term retention of “best practice” skills lab training (BPSL) versus a traditional “see one, do one” bedside teaching (TRAD) in a simulated setting of two different procedural skills (nasogastral tube insertion, NGT, and i.v. cannulation, IVC) at undergraduate medical educational level. Performances were assessed twice for each student: immediately after training and at 3 or 6 months follow-up, respectively.

### Sample size

A power analysis was undertaken to determine the necessary sample size. An effect size according to Cohen’s d = 1.2 was expected from training assessment data obtained from our previous studies [28,29]. For this study, we aimed at a power of ≥ 0.8.

### Skills classification

The complexity of the skills was determined by 10 expert interviewers who rated them on a 10-point Likert scale (0= very easy to 10 = very complex). A rating below 5 was considered a simple skill. One simple and one more complex skill were chosen.

### Student sample and Randomization procedure

**Figure 1:**
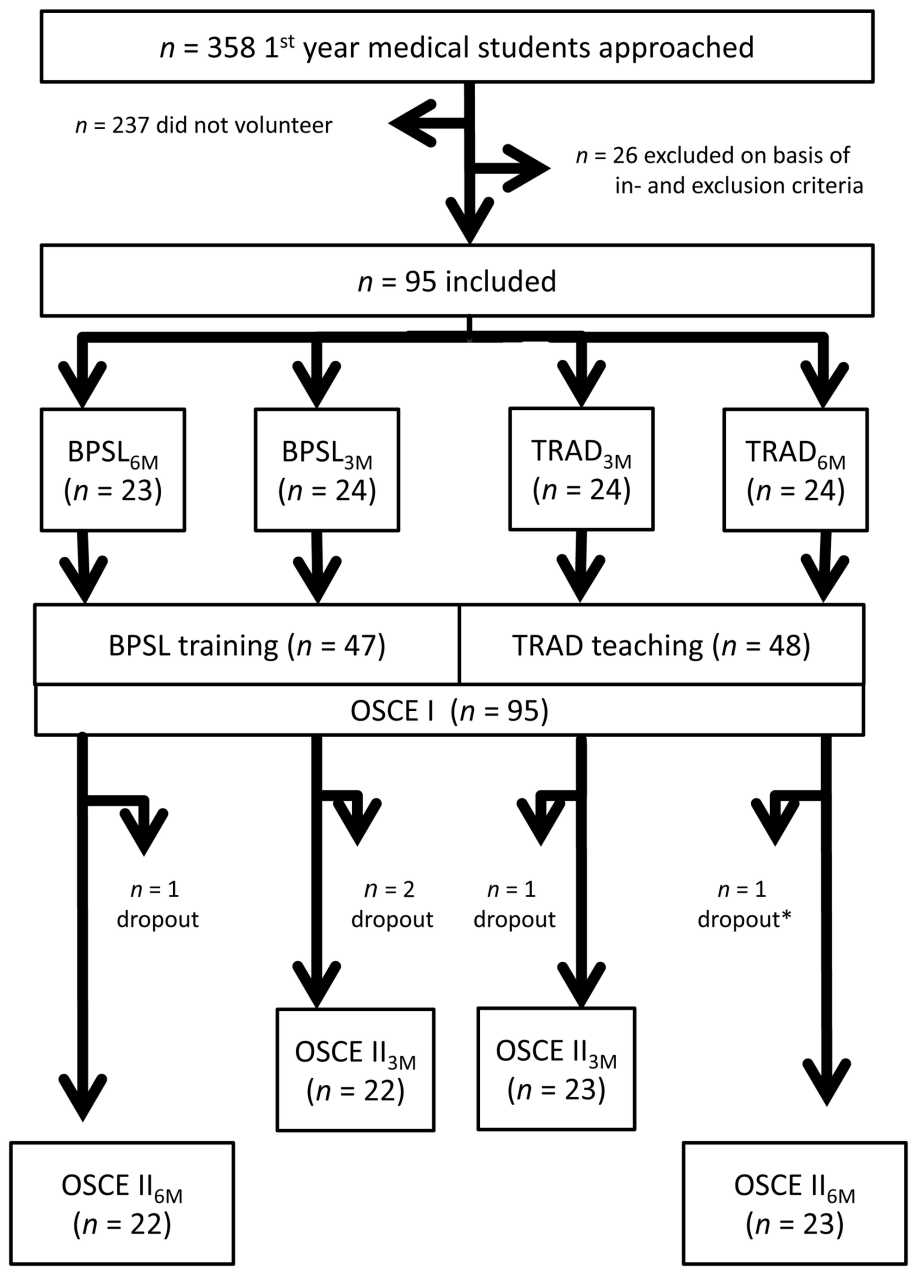
Study design and randomisation process. BPSL = “best practice” skills lab, TRAD = “see one, do one”, OSCE = Objective Structured Clinical Examination, 3M = follow-up after 3 months, 6M = follow up after 6 months * = video material not useable.

Inclusion criteria were: first year medical student at the University of Tübingen, within the first six months of training, and at the time of investigation not within any skills lab training. Students were excluded on the basis of the following criteria: previous training as a paramedic or nurse, prior experience in intravenous cannulation, urinary catheter or nasogastric tube insertion, and/or inability to attend the teaching sessions within the given timeframes. Therefore, the whole cohort of 1^st^-year medical students at the University of Tübingen in 2011 (n=358) was approached for participation in the study. The 95 voluntarily participating and eligible medical students were allocated by means of blocked randomisation to one of four groups: i) “best practice” skills lab training with follow-up after 3 months (BPSL_3M_: *n*=24), ii) “best practice” skills lab training with follow-up after 6 months (BPSL_6M_: *n*=23), iii) “see one, do one” with follow-up after 3 months (TRAD_3M_: *n*=24), and iv) “see one, do one” with follow-up after 6 months (TRAD_6M_: *n*=24). For details of study design and randomisation procedure see Figure 1.

### Pre-assessment

Before the first teaching session, we recorded socio-economic and educational background data to ensure that there were no confounders among the four groups. In addition, the students’ general learning strategies were characterised by two standardized questionnaires: The Kolb Learning Style Inventory (LSI) [30,31] and the General Self-Efficacy Scale (GSE) [32].

The Kolb Learning Style Inventory aims to define an individual’s specific learning preference. It consists of 12 items ranked on a four-point Likert scale (4 = most like me to 1= least like me) and results in one out of four learning modules: Concrete Experience (CE) - Abstract Conceptualization (AC) - Reflective Observation (RO) - Active Experimentation (AE) [30,31].

The General Self-Efficacy Scale is also ranked on a four-point Likert scale (1 = I agree to 4 = I disagree) and assesses 10 items with regards to perceived self-efficacy [32].

Both LSI and GSE have been shown to provide a reliable measurement for students’ learning motivation and self-assessment; these two parameters were considered potential confounder in addition to former education or clinical practice [33,34].

### Conceptual frameworks

As conceptual frameworks for the learning content we used standard up-to-date manuals, which have been used regularly in our classes as well as for our previous studies [35,36].

Regarding the conceptual frameworks for methods, the current study was based on Ericsson’s model of deliberate practice with feedback as the basis of our skills lab training, and Bandura’s social learning theory as basis for the traditional bedside teaching [37,38].

### “Best practice” skills laboratory training

The two intervention groups (BPSL_3M_: *n*=22 and BPSL_6M_: *n*=22) trained both procedural skills (NGT and IVC) in the skills laboratory using structured individual feedback, performance on manikins and Peyton’s “Four-Step-Approach” [39,40] which consists of the following four steps: 1. The teacher demonstrates the skill at his normal speed without any comments (“Demonstration”). 2. The teacher repeats the procedure, this time describing all necessary sub-steps (“Deconstruction”). 3. The student has to explain each sub-step with the teacher following the student’s instructions (“Comprehension”). 4. The student performs the complete skill on his own (“Performance”). Each student was allowed to perform step 4 once for each skill. Each session was held in a group of three students with one teacher. The “Life/form Adult Venipuncture and Injection Training Arm”, a part-task-trainer model shaped like a human arm, was used for intravenous cannulation (Nasco, Fort Atkinson, USA). The “Nursing training manikin CLA 1+8” (CLA, Coburg, Germany), a whole body model, was manikin for nasogastric tube insertion. Both skill trainings were embedded into a clinical scenario with role-play to create a more realistic training situation and to enhance the students’ involvement [4,41]. Afterwards, students received feedback about their performance. The student:teacher ratio was 3:1.

### “See one, do one” teaching

The two other teaching groups (TRAD_3M_: *n*=23 and TRAD_6M_: *n*=24) received training according to the well-known teaching principle of “see one, do one”, commonly part of bedside teaching, within a simulated setting. In order to control for time differences arising due to practice in BPSL training, a thorough theoretical introduction on both procedures, NGT and IVC, was given to the students. This was followed by the practical part where the teachers showed both skills on the same manikins within the same skills laboratory setting as in the intervention groups. They explained each step while they were performing it. Students were asked to watch the performance attentively and were allowed to make themselves familiar with the material used but were not allowed to practise the skills within the teaching session itself. As with the “best practice” skills lab group, the student:teacher ratio was 3:1.

### Skills lab setting and teachers

All teaching took place within DocLab, the skills lab of the Medical Faculty of Tübingen. This was done to make the teaching conditions as comparable as possible and to control for a maximum of possible confounders. At any given time, only one method (BPSL or TRAD) was taught to avoid cross-contamination. In total, 8 teachers ran the training sessions. All teachers were experienced student tutors of our skills lab whose equality in teaching performance as compared to faculty staff has been shown previously [29]. They were randomised to one of the teaching methods. Additionally, the teachers received thorough instructions for their respective teaching session and a detailed manual including defined learning goals, a comprehensive teaching agenda and detailed information about the time available for each section of the teaching session. Intervention group teachers received a refresher in Peyton’s “Four-Step-Approach” [39,40]. All teachers were blinded to the study design and only taught one method (BPSL or TRAD) to avoid any reciprocal interference in teaching style.

### Assessment of trained skills

After the teaching sessions, we immediately videotaped the students’ performance at two assessment stations (nasogastric tube insertion, i.v. cannulation) on the same mannequins as used in training comparable to the ones used in OSCEs (Objective Structured Clinical Examination). Each student was alone in the assessment room and had only one attempt to perform the skill learned. The other students from the corresponding teaching group waited in a different room with a supervisor present until it was their respective turn for individual assessment. The total amount of time needed was recorded for each skill at both assessments dates. Two blinded video-assessors rated the performance according to pre-defined binary and global checklists.

According to group randomisation, we re-invited students after either 3 or 6 months. This time, they did not receive any teaching but were asked to perform both skills again on the same manikin in the same environment as before. Once more, students were assessed alone according to the method (BPSL or TRAD). There was an assistant present to take care of any students who arrived before their allocated assessment time. Again, students were videotaped to be rated by video-assessors via the identical checklists. All students signed an agreement not to talk to their fellow students about their experienced teaching method and not to practise the skills in between assessment date one (OSCE I) and two (OSCE II, see Figure 1).

### Video rating

For videotaping, high-resolution cameras with optical zoom were used. Afterwards, all videos were digitally processed and randomised in regard to the playing sequence so that no conclusions could be drawn from it. Two blinded video assessors evaluated students’ performance according to a binary checklist and a global rating form, both already used in our previous studies [28,29,42]. Video assessors were encouraged to fill in the boxes as soon as students showed the respective step. The single-item global rating on a six-point Likert-scale (6 = very good to 1 = unsatisfactory) referred to the overall performance and was given at the end of intravenous cannulation or nasogastric tube insertion. It was furthermore categorised into “competent students” (rated as ‘6’ and ‘5’), “borderline students” (rated as ‘4’ and ‘3’) and “incompetent students” (rated as ‘2’ and ‘1’).

### Statistical analysis

All data are presented as means ± standard error of the mean (SEM), if not otherwise stated. Data were tested for normal distribution using the Shapiro-Wilk-Test. Normally distributed data were compared using a Student’s t-test (assuming equal variances). A Mann-Whitney U-Test (MWU) was used for non-normally distributed parameters. For reader’s convenience, results of the MWU tests are not displayed as sum of ranks. The distribution of discontinuous group characteristics was compared by the chi-square test. Effect sizes were calculated using Cohen’s d for continuous variables and Cramer’s φ for 2-level associations. Standardized inter-rater reliability for the two video assessors was calculated based on Kappa analysis. A *p*-value <0.05 was considered to be statistically significant. Raw data were processed using Microsoft EXCEL (Microsoft Inc., Redmond, WA, USA). The software packages JMP (SAS Institute Inc., Cary, NC, USA) and SPSS (SPSS Inc., Chicago, IL, USA) were used for statistical analysis.

### Ethics

Study participation was voluntary and all students were assured of anonymity and confidentiality. Students were informed that the purpose of the study was the comparison of different ways of teaching but they did not receive any details. The ethics committee of the University of Tübingen waived the requirement of further ethical approval based on the condition that all data were analyzed anonymously (Nr. 539/2012A and 296/2008A). However, written consent was obtained from all students.

## Results

### Sample size

Based on our preliminary studies, power analysis showed that *n*=15 students were needed for each of the four groups to detect the expected effect size (α=0.05; power 0.8). Of the 358 students approached, 121 medical students volunteered to participate. After initial screening according to our criteria, 95 were eligible to be included in the study. Three students failed to attend follow-up and another video was not assessable for technical reasons, therefore the final number of students whose performance could be completely assessed was 91 (BPSL_3M_: *n*=22, BPSL_6M_: *n*=22, TRAD_3M_: *n*=23, TRAD_6M_: *n*=24).

### Student sample

All participating students were in their first year. The average age of the complete study cohort was 21.4±0.5 years. A total of 26 out of 91 students were male. With two exceptions (gender distribution between BPSL_3m_ and TRAD_3m;_ prior practical nursing days between BPSL and TRAD at baseline), there were no significant differences between the four randomized groups regarding socio-demographic variables, former health care education, previous clinical experience or scores in the above described standardized questionnaires LSI [30,31] and GSE [32] as shown in table 1 (all p>0.06).

**Table 1:** Basic socio-demographic characteristics of participants.

**Sociodemographic variables**	**BPSL 3M (n=23**)	**BPSL 6M (n=23**)	**TRAD 3M (n=22**)	**TRAD 6M (n=23**)	**ANOVA**	**p_1_**	**p_2_**	**p_3_**
**Gender** [male/female]	5/18	6/17	8/14	7/16	-	0.32^a^	**0.02**	0.74
**Age** [years]	20.9 [19.8;22.0]	21.6 [19.5;23.8]	20.2 [19.5;21.0]	22.9 [19.9;25.8]	.23	0.38^b^	0.75	0.13
**Prior HealthCare Education** [yes/no]^1^	4/19	3/20	2/20	2/21	-	0.35^a^	0.41	0.64
**Prior Study** [yes/no]^2^	5/18	3/20	4/18	6/17	-	0.56^a^	0.77	0.26
**Practical Nursing** [days]	22.3 [7.1;37.5]	41.6 [26.2;57.1]	45.7 [28.5;62.8]	55.4 [37.5;73.3]	0.07	**0.04** ^b^	0.06	0.21
**Prior Blood Sampling Procedures** [yes/no]	0.7 [0;1.4]	2.2 [0.3;4.1]	2.6 [0;7.4]	0.3 [0;0.5]	.35	0.35^b^	0.71	0.10
**Prior Nasogastric Tube Procedures** [yes/no]	0	0	0	0	-	1^b^	1	1
**Prior Intravenous Cannulation Procedures** [yes/no]	0	0	0	0	-	1^b^	1	1
**Handedness** [left/right]	1/22	1/22	2/20	2/21	-	0.40^a^	0.55	0.55
**Questionnaires**	**BPSL 3M (n=23**)	**BPSL 6M (n=23**)	**TRAD 3M (n=22**)	**TRAD 6M (n=23**)	**ANOVA**	**p_1_**	**p_2_**	**p_3_**
**General Self-Efficacy Scale** [40-10] [32]	20.0 [18.4;21.7]	21.0 [18.6;23.4]	19.3 [16.4;22.2]	20.4 [17.6;23.1]	0.76	0.88^c^	0.64	0.73
**Kolb LSI** [10-40] [30,31] Abstract_Conceptualization Concrete_Experience Active_Experimentation Reflective_Observation	32 [0;47] 25 [0;36] 35 [0;44] 29 [0;39]	31 [0;43] 21 [0;40] 32 [0;44] 28 [0;40]	34 [0;46] 22 [0;36] 34 [0;43] 31 [0;40]	32 [0;43] 23 [0;40] 33 [0;45] 28 [0;41]	0.82 0.58 0.89 0.63	0.81^b^ 0.76^b^ 0.72^b^ 0.43^b^	0.87 0.25 0.55 0.13	0.81 0.72 1 0.77

All data are presented as means with the 95% confidence intervals provided in square brackets, except the results from the Kolb Learning Style Inventory (LSI) which are shown as medians [min; max].

^1^Prior HealthCare education included: biological technical assistant, biologist, medical technical assistant, physiotherapist, social care worker, surgical technologist.

^2^Prior Study included: teaching, psychology, biology, business administration, business informatics, chemistry, engineering, law, molecular biosciences, pharmacy, and physics.

P-values p_1_, p_2_ and p_3_ refer to the following comparisons:

p_1_: TRAD (3M and 6M pooled together)” and “BPSL (3M and 6M pooled together)”

p_2_: TRAD 3M vs. BPSL 3Mp_3_: TRAD 6M vs. BPSL 6M, using ^a^ Chi^2^-test or ^b^ Mann-Whitney-U-Test.

BPSL = “best practice” skills lab teaching, TRAD = traditional “see one, do one” teaching, 3M = assessed 3 months after training, 6M = assessed 6 months after training, ANOVA = analysis of variance

### Skills complexity

Skills rating by experts for NGT was 1.7±1.1 and for IVC 6.5±1.1.

### Teaching sessions

Length of teaching sessions did not differ significantly between the four different teaching groups (BPSL_3m_ 89.6±1.0 min, BPSL_6m_ 89.5±0.8 min, TRAD_3m_ 89.9±0.7 min, TRAD_6m_ 89.7±0.8 min, p_ANOVA_=0.58).

### Video-rating

#### Time needed for performance of skills

Time was measured from picking up the first item until the student announced the end of the procedure. There was a significant difference between the BPSL and the TRAD group performance time at t_0_, measured immediately after teaching, for both NGT (TRAD_3M/6M_ 335±20 sec, BPSL_3M/6M_ 294±16 sec, p<0.001) and IVC (TRAD_3M/6M_ 657±52 sec, BPSL_3M/6M_ 522±36 sec; p<0.001). The significant difference in favour of a lower performance time needed in the BPSL group remained stable at each respective long-term assessment for both skills (data not shown).

#### Assessment at examination stations by binary checklists

The number of correctly performed steps for nasogastric tube insertion and i.v. cannulation identifiable on the video tapes was calculated as the percentage of maximal achievable binary checklist points (NGT: 26 points, IVC: 29 points). Immediately after teaching, students from our intervention groups trained via “best practice” skills lab teaching scored significantly higher at performance of NGT insertion (BPSL_3M_ 94.8%±0.2, BPSL_6M_ 95.4%±0.3) and IVC (BPSL_3M_ 86.4%±0.5, BPSL_6M_ 88.0%±0.5) than the comparison group receiving only teaching according to the traditional “see one, do one” approach (NGT: TRAD_3M_ 86.1%±0.5, TRAD_6M_ 84.7%±0.4 and IVC: TRAD_3M_ 73.2%±0.7, TRAD_6M_ 72.5%±0.7). According to these ratings, BPSL resulted in an effect size (Cohen’s D) of 1.32/1.36 (NGT/IVC) and 1.57/1.64 (NGT/IVC) after 3 and 6 months, respectively, compared to TRAD.

**Figure 2:**
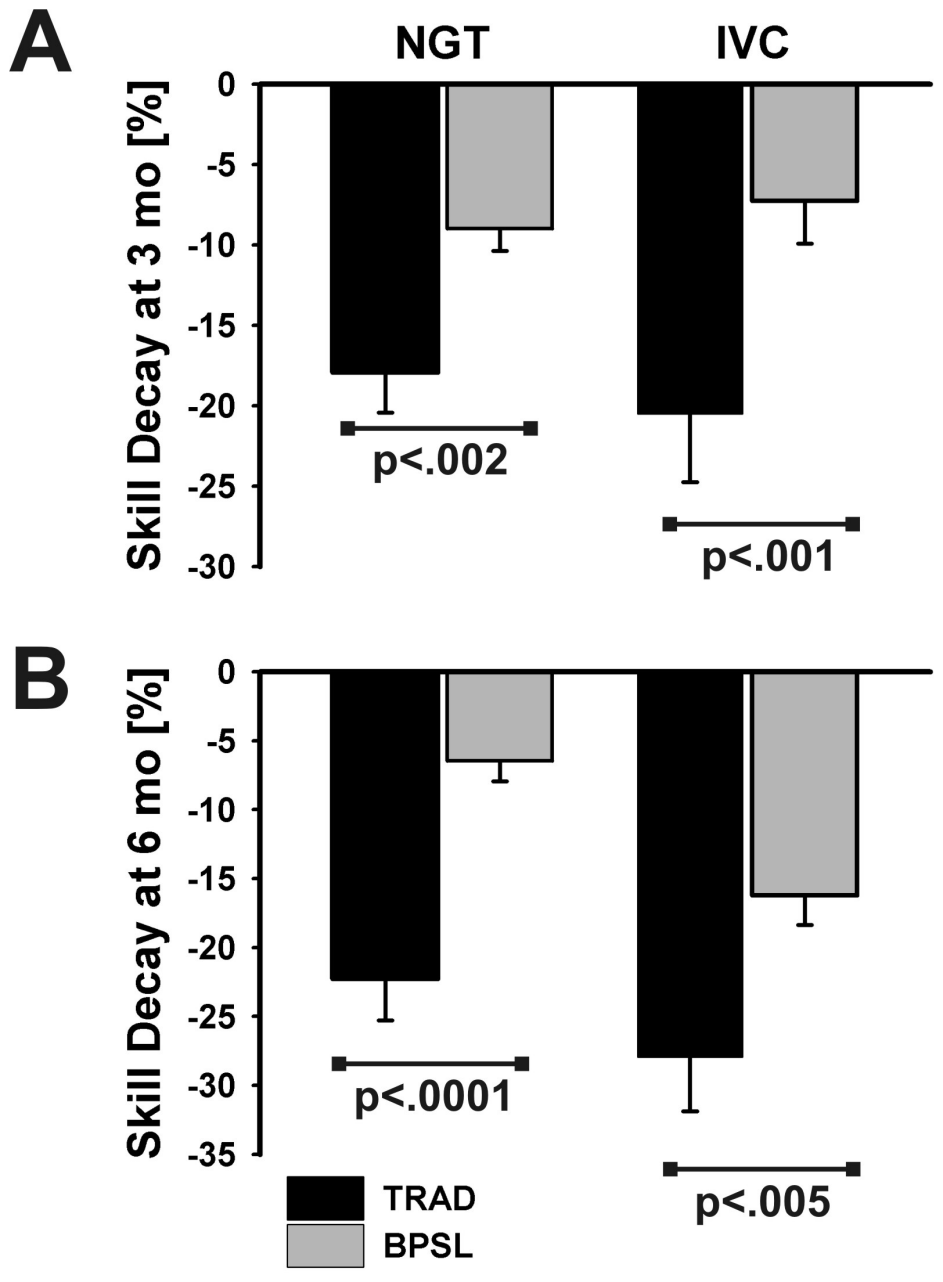
Loss of skills level expressed as percentage of points on the corresponding binary checklist (NGT or IVC) at 3 (Figure 2A) and 6 months (Figure 2B) after initial training, respectively (error bars refer to SEM). NGT = nasogastral tube insertion, IVC = intravenous cannulation, BSPL = Best Practice Skills Lab Training, TRAD = Traditional “see one, do one” teaching.

Within each group, there was a significant loss of performance when tested after 3 months or 6 months respectively. The obtained percentage scores according to the binary checklists of all participants at the first (t_0_) and respective second assessment station (t_1_ after 3 or 6 months, respectively) are shown in Table 2. The corresponding percentages and p-values of skill decay according to each skill and time of assessment are depicted in Figure 2. BPSL students lost significantly less of their performance than their TRAD fellows at all times of assessment (see Figure 2).

**Table 2:** Percentages of maximal achievable points with the 95%CI provided in squared brackets on binary checklist (NGT = 26 points, IVC = 29 points).

	**t_0_ (immediately after teaching**)				**t_1_ (after 3 months**)			
	**TRAD 3M**	**BPSL 3M**	**Cohen D^1^**	**p^2^**	**TRAD 3M**	**BPSL 3M**	**Cohen D^1^**	**p^2^**
NGT	86.1% [82.4;89.8]	94.8% [93.2;96.6]	1.32	<0.0001	70.3% [65.9;74.7]	86.3% [83.6;88.9]	1.59	<0.0001
IVC	73.2% [68.4;78.0]	86.4% [83.0;89.7]	1.36	<0.0001	56.5% [53.2;59.8]	79.5% [75.9;83.0]	2.87	<0.0001
	**t_0_ (immediately after teaching**)				**t_1_ (after 6 months**)			
	**TRAD 6M**	**BPSL 6M**	**Cohen D^1^**	**p^2^**	**TRAD 6M**	**BPSL 6M**	**Cohen D^1^**	**p^2^**
NGT	84.7% [81.3;88.1]	95.4% [93.1;97.7]	1.57	<0.0001	65.4% [60.4;70.4]	89.0% [86.9;91.1]	2.64	<0.0001
IVC	72.5% [67.7;77.2]	88.0% [84.7;91.4]	1.64	<0.0001	51.5% [45.7;57.3]	73.2% [70.6;75.8]	2.07	<0.0001

3M = follow-up after 3 months, 6M = follow-up after 6 months. NGT = nasogastral tube insertion, IVC = intravenous cannulation. BPSL = “best practice” skills lab teaching, TRAD = traditional “see one, do one” teaching.

^1^Cohen’s d was calculated using means and standard deviations of achieved binary checklist points. BPSL (TRAD) was considered as treatment group (control group).

^2^p-values were calculated using the Mann-Whitney-U test on ranks.

#### Assessment at examination stations by single-item global rating

**Figure 3:**
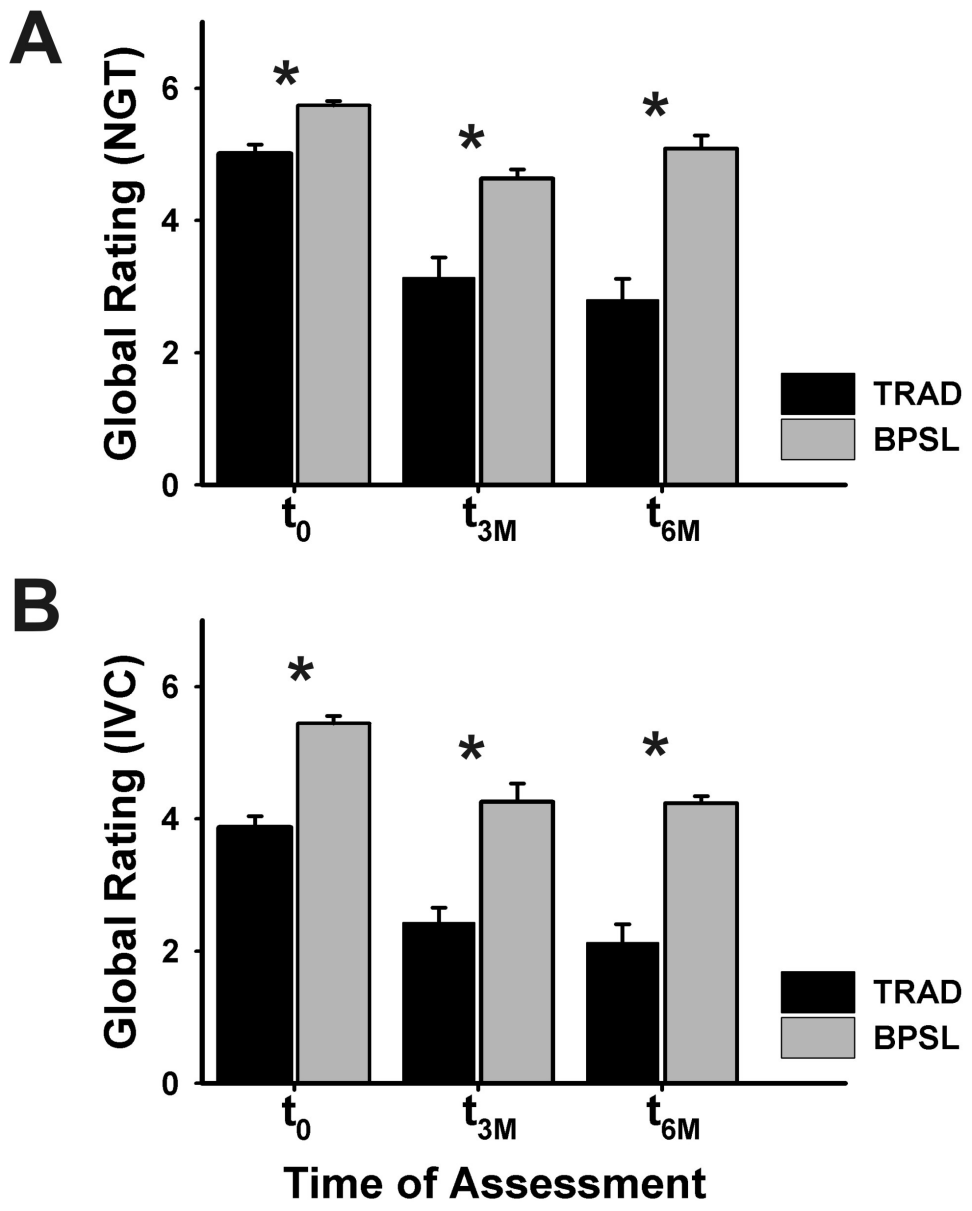
Single-item global rating of performance (mean + SEM) initially after training (t_0_) and at 3 (t_3M_) and 6 months (t_6M_) later, respectively. NGT = nasogastral tube insertion, IVC = intravenous cannulation, TRAD = traditional “see one, do one” training, BPSL = Best Practice Skills Lab Training. Significant differences (p<0.05) are marked with an asterisk.

The single global item “Overall ability to perform the procedure” showed similarly, that the BPSL group was rated more often clinically competent than the TRAD group for both tested skills (NGT, IVC) at all times of assessment (t_0_ immediately after teaching, t_3M_ after 3 months and t_6M_ after 6 months; see Figure 3). The categorisation into “competent”, “borderline”, and “incompetent” particularly showed the superiority of BPSL students (see table 3). NGT, as a clinical skill of lower complexity experienced a slightly greater benefit from BPSL training than IVC representing a task of higher procedural complexity.

**Table 3:** Global rating categorised into 1 = “competent students”, 2 = “borderline students” and 3 = “incompetent students”.

**NGT**						
	t_0_	t_1_	t_2_	p_1_ (Cramer’s ϕ)	p_2_ (Cramer’s ϕ)	p_3_ (Cramer’s ϕ)
**BPSL**	1 = 45 (97.8) 2 = 1 (2.2) 3 = 0 (0.0)	1 = 13 (56.5) 2 = 10 (43.5) 3 = 0 (0.0)	1 = 19 (82.6) 2 = 3 (13.0) 3 = 1 (4.4)	0.0008 (0.35)	0.0003 (0.60)	<0.0001 (0.70)
**TRAD**	1 = 33 (73.4) 2 = 12 (26.6) 3 = 0 (0.0)	1 = 2 (9.1) 2 = 12 (54.5) 3 = 8 (36.4)	1 = 3 (13.0) 2 = 10 (43.5) 3 = 10 (43.5)			
**IVC**						
	t_0_	t_1_	t_2_	p_1_ (Cramer’s ϕ)	p_2_ (Cramer’s ϕ)	p_3_ (Cramer’s ϕ)
**BPSL**	1 = 41 (89.1) 2 = 5 (10.9) 3 = 0 (0.0)	1 = 11 (47.8) 2 = 10 (43.5) 3 = 2 (8.7)	1 = 5 (21.7) 2 = 18 (78.3) 3 = 0 (0.0)	<0.0001 (0.60)	0.0001 (0.63)	<0.0001 (0.77)
**TRAD**	1 = 14 (31.1) 2 = 25 (55.6) 3 = 6 (13.3)	1 = 1 (4.6) 2 = 7 (31.8) 3 = 14 (63.6)	1 = 1 (4.4) 2 = 5 (21.7) 3 = 17 (73.9)			

t_0_ = immediately after teaching, t_1_ = after 3 months, t_2_ = after 6 months. Percentages are shown in round brackets. NGT = nasogastral tube insertion, IVC = intravenous cannulation.

BPSL = “best practice” skills lab teaching, TRAD = traditional “see one, do one” teaching.

P-values were calculated using Chi^2^-test. p_1_: TRAD_(3months and 6months pooled together)_ vs. BPSL_(3months and 6months pooled together)_, p_2_: TRAD_3months_ vs. BPSL_3months_, and p_3_: TRAD_6months_ vs. BPSL_6months_. The effect sizes were calculated using Cramer’s phi.

### Inter-rater reliability

Standardised inter-rater reliability ranged from 0.734 to 0.870 (p<0.001) for binary checklists and 0.911 to 0.931 (p<0.001) for the single-item global rating indicating a good to very good agreement.

## Discussion

This study prospectively investigated the long-term retention of two different skills (nasogastric tube insertion; intravenous cannulation) taught in two different ways (“best practice” skills lab training and a “see one, do one” approach) to first year medical students. Following assessment after training, students were re-invited for a second assessment either 3 or 6 months after initial training sessions according to randomisation. Students were carefully selected according to in- and exclusion criteria and randomized to one of the four groups. There were no significant differences in socioeconomic background and other potentially influencing variables among the four different study cohorts. Students who received the “best practice” model of skills lab training showed significantly better results measured with binary checklists as well as a single-item global rating at measurement immediately after teaching (T_0_) and each follow-up (3 or 6 months, respectively). Interestingly, BPSL training led to better assessment results, in particular for IVC which represents a task of higher complexity, while NGT as a lower complexity skill made its benefit from BPSL training primarily in regard to its long-term retention (see Figure 3). It should be emphasized that the resulting effect sizes attributed to BPSL can be considered quite large. This was surprising given the long time interval between the assessments (3 and 6 months, respectively) and the short intervention time (90 min each for both BPSL and TRAD teaching sessions).

Being trained in the “best practice” skills lab group also led to a significantly shorter time needed for performance of both skills, despite the fact that both tested teaching methods occupied the identical resources in teaching time. Furthermore, BPSL students were significantly more often rated as “competent” than TRAD students.

To our knowledge, the long-term retention of skills taught to medical students comparing a “best practice” model of skills lab training and a “see one, do one” approach as frequently used in clinical bedside teaching has not been investigated so far. Most of the research contrasting simulation-based medical education (SBME) and traditional teaching methods to date has focused on residents, and was either concerned with complex surgical or intensive care procedures [43,44] or refers to cardiac life support training [18]. It is well known, that straight after training SBME with “deliberate practice” is superior to traditional clinical teaching in the acquisition of a broad variety of skills [38,45]. We could show that on top of the immediate effect, there is a long term benefit of “best practice” skills lab training with structured individual feedback, training on manikins and Peyton’s “Four-Step-Approach” improves students’ performance in a relatively simple task (nasogastric tube insertion) as well as a more complex one (intravenous cannulation). There is a relative superiority of BPSL after 3 or 6 months and a smaller loss of correctly performed steps in absolute terms.

Within the “best practice” skills lab training, various factors contributed to the teaching: First, BPSL teachers acted as role models when showing the skill, helping students to observe the correct procedure. Secondly, we included Peyton’s “Four-Step-Approach” [22], which implies a rather unusual step, namely instructing the teacher step-by-step how to perform the skills. This comprises the necessity to review all steps on their own and also reflect upon the procedure itself. Reflection has been shown to be a crucial step in memory consolidation [46]. Additionally, BPSL students had the advantage of “learning through teaching” by instructing someone else in step 3 of Peyton’s “Four-Step-Approach”. If students made a mistake, they could see the immediate implication as the tutor followed all instructions given by the student regardless of them being correct or not. This gives immediate feedback and allows students to monitor their progress. This kind of metacognitive awareness is a hallmark feature of self-regulated learning, which is an approach that actively incorporates students into the learning process [47]. Students are encouraged to show initiative and take responsibility for what and how they learn [48]. Particularly in its directed form as described by Brydges and colleagues, self-regulated learning can lead to superior long-term performance when compared to an instructor-regulated approach [49]. This could help to explain why our results do not seem to be in line with the “performance–learning paradox”, which refers to the common finding that immediate performance can be quite good but does not necessarily reflect long-term learning [50]. In the current study, students trained in skills labs were not only better at immediate assessment but also at the respective follow-up assessment after 3 or 6 months, respectively. As another component of the “best practice” skills lab training, BPSL students received structured individual feedback on their own performance. This is well recognised to represent a key feature of effective skills lab training [5]. Simulators alone are not enough to improve skill performance [51]. Feedback helps medical students to get a feeling for what they do and increases the likelihood of correct performance [52]. On the other hand, research in motor learning shows that feedback from instructors improves immediate performance but can be a hindrance for long-term learning [53]. In our study this was not supported, as the “best practice” skills lab teaching group had more feedback but performed better in the long run. This might be due to other components in line with the concept of self-regulated learning like review at each stage and self-monitoring as described above. Finally, by integrating the aspect of role-play into BPSL teaching, a more realistic training scenario was created [4].

On the contrary, the alternative teaching followed the “see one – do one” principle, usually common in clinical bedside teaching. TRAD Students’ only way of learning the skill was through attentive observation as in line with the social learning theory [37]. Tutors acted as role models by demonstrating each skill to the students. However, all other components described as part of skills lab teaching were completely missing, and students never had the opportunity to practise the skill before the first assessment, as this is not part of a traditional bedside situation. This might explain their inferiority to BPSL students.

Nonetheless, in general, there was a decline in the ability to perform both skills in nearly all students. Only 7 students out of the 91 (3 BPSL, 4 TRAD) showed an improvement from t_0_ to their respective long-term follow-up. This is likely explained by the fact that they were all amongst the bottom performers at baseline point and, thus profited by the training effect of a 2^nd^ assessment.

We added a global rating to the binary checklist in order to balance for the fact that a checklist ranks all items in the same way, potentially leading to failure because of several less important items rather than one big mistake [54]. Our good inter-rater reliability for both measurements validates our methodological approach to measure skill performance. Additionally, global ratings take into account the difference between competence shown in an assessment situation and performance shown under real life circumstances as described by Rethans et al. [55]. Furthermore, we tried to improve the validity of our skills training and testing by creating a role-play scenario as validity improves effective learning [4,56].

We deliberately used student tutors as teachers who are teaching in our official classes within the curriculum. They have been shown to be equally effective and accepted as staff [29,57] and student tutors are meanwhile part of most faculties’ skills lab training [58]. Additionally, all tutors participating in our study received an elaborate manual as well as a training session before teaching the students.

In line with well-matched skills training, most students are being taught simple as well as more complex skills early on from the beginning of their studies. This is the first step of a process building up to the transfer of these skills to actual patient care. Following the results of our study, some recommendations for skills teaching can be made: Skills lab training comprising different teaching elements like structured individual feedback, practise on manikins and Peyton’s “Four-Step-Approach” should be an inherent part of undergraduate medical education. To optimise long-term outcome, these skills should be refreshed in at least biannual intervals for more complex skills, and annually for easier tasks. Further studies have to examine whether supervised practice outside regular curricular activities can also serve this purpose.

## Limitations

Several limitations of our study should be mentioned. As the study was done on a voluntary basis, there is of course the possibility of a selection bias with only the very motivated students showing up. However, there were a lot more students registering in the first place that had to be excluded due to exclusion criteria or time incompatibilities. Additionally, careful randomisation of all applicants to one of our four groups was performed.

Furthermore, we cannot exclude that some students practised the tasks on their own, although all students signed an agreement not to do so and additionally gave oral confirmation on their second testing day about it. In any case, it is very unlikely that they had a structured training in the meantime. Another limitation is the fact that all teaching took place in a simulated environment. This limits the generalizability of our findings with regards to clinical context. Additionally, students taught within the skills lab could practise each skill one time before doing the actual test in the assessment stations as opposed to bedside teaching students who had only seen someone else performing the task. However, this was part of one of the components included into the skills lab teaching, namely Peyton’s “Four-Step-Approach”. At the same time, the traditional “see one, do one” approach without this possibility to practise has been recognised as an appropriate teaching method, too [59]. Additionally, we made all other conditions like time or student to tutor ratio the same for both groups. Another limitation may be seen in that we did not undertake any assessment of students’ prior abilities. We deliberately waived this possibility during designing the study in order to minimize any training effect due to repetitive testing.

## Conclusions

In summary, we could show that a best practice skills lab training of intravenous cannulation and nasogastric tube insertion skills is superior to the traditional “see one, do one” approach not only immediately after training, but also at 3 or 6 months follow up. This observed superiority applies for single steps of the procedures, time needed to perform the skills, and the global clinical impression. With regards to the long-term performance, skills lab teaching seems to be particularly helpful for the reproduction of easier skills.

In line with previous studies that showed superiority of skills lab training immediately after teaching and in transfer to real patients for the two skills [9,40], this study underlines the importance of skills lab training being an integral part of teaching students with respect to long-term performance. Regular fresher classes have to be provided when our students are supposed to show stable performances. We suggest a biannual interval for more complex tasks and an annual one for easier tasks.

Further studies should investigate whether these findings hold up when transferred to real patients. Additionally, research in this field should focus on clarification studies as suggested by Cook et al., asking for how and why skills lab training seems to be so superior to the traditional “see one, do one” training provided by bedside teaching [60].
